# The Deckled Incision: Study Protocol for a Randomized Controlled Trial

**DOI:** 10.2196/resprot.5491

**Published:** 2016-07-12

**Authors:** Apresh Singla, Sarah J Lord, Quan Ngo

**Affiliations:** ^1^ St Vincents Hospital Sydney Australia

**Keywords:** scar improvement, post-operative

## Abstract

**Background:**

Scar visibility is multifactorial and skin closure technique is thought to play an important role. It is an established principle in plastic surgery that Z plasties generally reduce scar contracture by breaking up the lines of tension in a wound. As an extension of this principle, it is postulated that irregular “deckled” skin incisions made during tumor excision would produce aesthetically superior scars.

**Objective:**

The primary objective of this study is to assess both the clinician and patient opinion of scar quality using the Patient and Observer Scar Assessment Scale (POSAS). Secondary objectives include the proportion of scars judged as good by the both the patient and clinician (less than or equal to 5 on the overall PSOAS scale), the number of adverse events, and the proportion of the scar visible at 1 meter.

**Methods:**

The deckling study will be a patient-blinded, simple randomized controlled trial (RCT) at a single center institution. The two groups will be equally allocated on a 1:1 ratio into the control and treatment arms. All patients greater than 18 years of age undergoing a plastic surgery procedure involving excision of skin lesions will be enrolled. Any patients requiring re-excision through the wound or undergoing injectable corticosteroid therapy will be excluded. A total of 500 patients will be enrolled. The patients will be followed-up at 1 week, 3 months, and 6 months post-operatively.

**Results:**

The study is expected to begin enrolment in August 2016. We anticipate that the deckling study group will have superior scar outcomes when compared to the straight line incision. From clinical experience this is especially true for lesions involving the face and in those areas of the skin that have undergone radiation therapy. The study will be funded by the Plastics and Reconstructive Surgery Department at St Vincent’s Hospital, Sydney, Australia. Ethics approval has been obtained for the study. Conclusion: We believe this will be an important study to assess a novel method to improve the appearance of post-operative scars. The deckling study is simple to master, can be applicable to almost any surgical procedure, and can have good generalizability to a large population cohort.

**Conclusions:**

We believe this will be an important study to assess a novel method to improve the appearance of post-operative scars. The deckling study is simple to master, can be applicable to almost any surgical procedure, and can have good generalizability to a large population cohort.

**Trial Registration:**

Australian New Zealand Clinical Trials Registry (ANZCTR): ACTRN12616000193471; https://www.anzctr.org.au/Trial/Registration/TrialReview.aspx?ACTRN=12616000193471 (Archived by Webcite at http://www.webcitation.org/6gmG8yf1A)

## Introduction

### Disease Background

Scar visibility is multifactorial and skin closure technique is considered to play an important role. It is an established principle in plastic surgery that Z plasties generally reduce scar contracture by breaking up the lines of tension in a wound. As an extension of this principle, it is postulated that irregular “deckled” skin incisions made during tumor excision would produce aesthetically superior scars. A previous unpublished pilot study at St Vincent’s Hospital was conducted to look at deckled incisions versus straight incisions on head and neck lesions in 47 patients. It showed a statistically significant (*P* <.001) smaller detectable scar length ratio with the deckled incision compared to straight line incisions [1]. There are a limited number of studies that describe the use of the deckled incision in literature and they report improved scar formation and reduced contracture rates [2-4].

### Study Rationale

The aim of the study is to assess and quote whether using the deckled incision improves post-operative scars compared to the standard straight line incision. We hypothesize that the deckled incision has superior scar outcomes. Previous studies have been underpowered and had a number of biases as they were not conducted in a randomized fashion. The deckling study will be a patient-blinded, simple randomized controlled trial (RCT) at a single center institution. The two groups will be equally allocated on a 1:1 ratio into the control and treatment arms.

## Methods

### Objectives

The primary objectives of the study are (1) to assess clinician opinion of scar quality using the observer component of the Patient and Observer Scar Assessment Scale (POSAS); and (2) to assess the patient’s opinion of scar quality and/or sensation using the patient component of POSAS.

The secondary objectives are (1) to assess the proportion of scars described as good by the clinician (determined by ≤5 on the overall component of the POSAS); (2) determine the number of adverse events overall and by individual components (namely dehiscence infection, keloids); and (3) calculate the percentage of the scar visible as a percentage of the total length at 1 month.

### Design

The deckling study will be a patient-blinded, simple RCT at a single center institution. The two groups will be equally allocated on a 1:1 ratio into the control and treatment arms. There will be two groups: those that receive the deckled incision and those that receive the standard straight line incision.

### Participants

The study will be solely undertaken at St Vincent’s Public Hospital. The care providers involved with the study will include plastics and reconstructive surgery consultants and registrars. A total of 500 patients will be recruited and divided evenly into the two treatment groups.

### Duration

The study will be conducted over a period of 24 months with ongoing recruitment or until the total sample size required is reached.

### Inclusion and Exclusion Criteria

Inclusion criteria for the study include (1) greater than or equal to 18 years of age; (2) able to give informed consent and willingness to participate in follow-up; and (3) undergoing any plastic surgery procedure involving excision of skin lesions. Exclusion criteria include those requiring re-excision through the original wound and undergoing injectable corticosteroid therapy into the scar.

The patients will be randomly allocated to receive either the deckled incision or the straight line incision for their surgery. In patients with multiple (two or more) separate lesions requiring separate incisions, each lesion will be randomly allocated to receive either the deckled or straight line incision. Only the treating surgeon will be aware of which incision was used and the patient will be blinded to the type of incision. The surgeon will document in the operation note the number and site of lesions, whether they were deckled or not, the precise length of the scar, and whether the procedure was carried out by a registrar or consultant.

**Figure 1:**
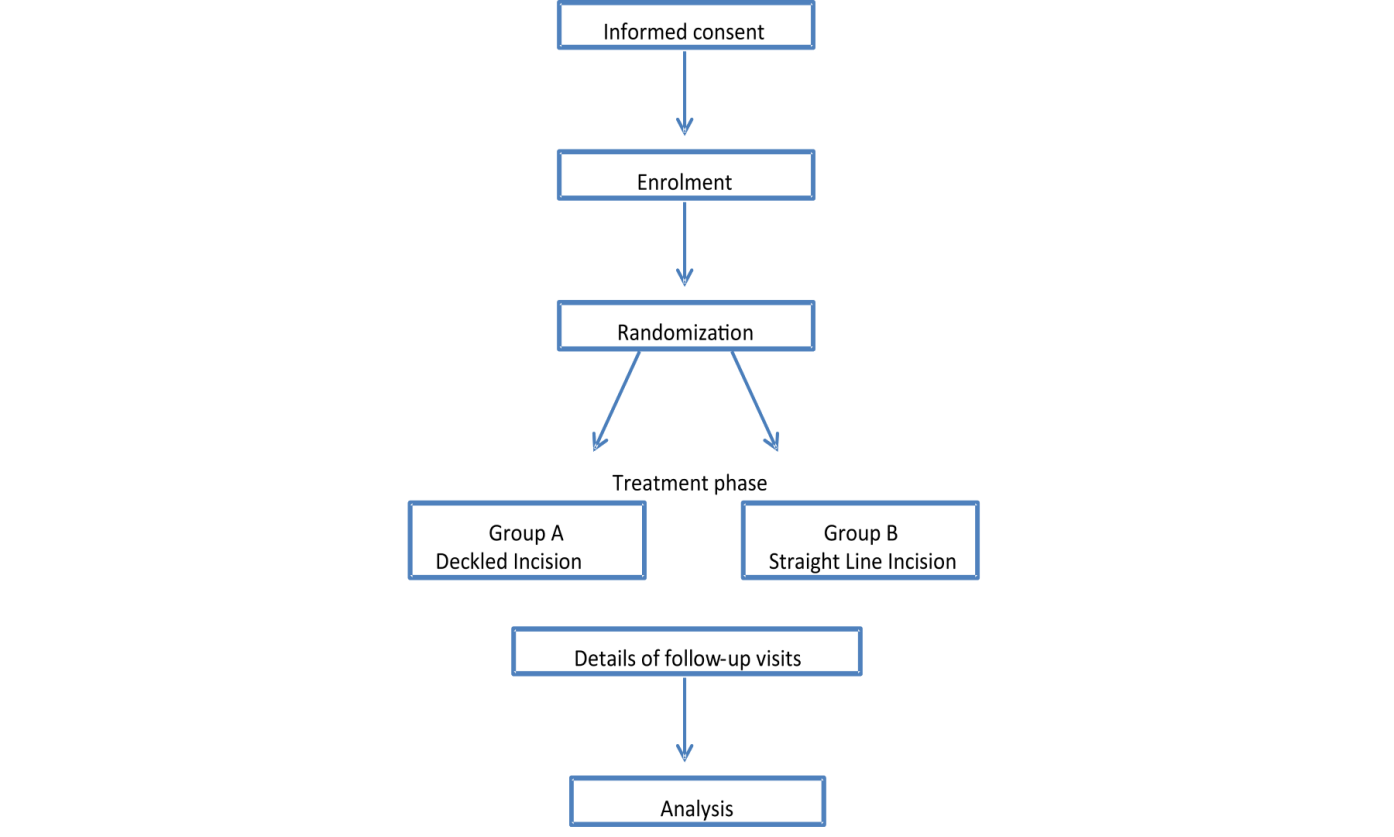
Study flow chart.

The patient will be followed-up in the outpatient clinic. Standard post-operative care with micropore tape will be given to both groups with respect to scar minimization. The scar and compliance with standard post-operative scar reduction will be assessed at 1 week, 3 months, and 6 months post-operatively using the POSAS [5]. The 6-month cut off was chosen as scars have usually matured by 6 months to reflect their final life-long appearance and to avoid a large number of patient attrition over a longer follow-up period (Figure 1).

### Investigation Plan

The intervention plan is shown in Table 1.

**Table 1:** Intervention plan.

List interventions	Enrolment visit	1 Week	3 Months	6 Months
Informed consent	✓			
Inclusion/exclusion criteria	✓			
Medical history	✓			
Patient and observer scar assessment scale (POSAS)		✓	✓	✓
Adverse event and serious adverse event assessment		✓	✓	✓

The follow-up will be done in the outpatient setting. The POSAS requires at least three independent assessors to achieve a valid result and this will be done by a combination of medical and nursing staff. The results will be recorded in real-time on a tablet (iPAD), which has been pre-populated with the patient demographics and the unique study code identifier that can be cross referenced with the medical records. The data is automatically backed up to an online database via Dropbox. Selected photographs will be taken by a digital camera for future publication purposes.

The routine standard of follow-up will vary with each individual procedure and thus the above protocol is not the standard of care. The additional costs of the procedure will be covered by the Plastics and Reconstructive Surgery Department.

### Study Procedure Risks

We anticipate minimal additional risks by undertaking the study. The deckled incision is fairly common practice among the Plastic Surgery Department and as previously illustrated in the pilot study, all other aspects of the post-operative care are standard.

### Recruitment and Screening

The patient will be recruited from outpatient and inpatient referrals and the Emergency Department at St Vincent’s Public Hospital.

### Informed Consent Process

Once the patient is identified as suitable, informed consent will be obtained to be enrolled in the study. The patient will be explained how the procedure is conducted, the expected post-operative recovery time, and the follow-up time periods. The risks and benefits of the procedure will be explained and the patient will be made aware of suitable alternatives [6].

### Enrolment Procedure

The participant will be enrolled into the study after the informed consent process has been completed, and the participant has met all inclusion criteria and none of the exclusion criteria. The patient’s baseline characteristics will be recorded on a pre-populated A4 sheet and scanned via email to a single email address. The patient baseline characteristics that will be recorded are age, gender, smoking, Fitzpatrick classification of skin type and ethnicity, presence of vascular co-morbidities, diabetes, use of systemic and/or oral steroids or immunosuppressant’s, previous poor scar results, site of lesions (face vs trunk vs limbs), and previous radiotherapy over the surgical site. An independent person will subsequently enter the patient data into an online database and allocate a study code, which will be documented in the patient records.

The final statistical analysis will be stratified and adjusted by the patients’ baseline characteristics to adjust for their effects on wound healing. We are particularly interested in effects of previous radiotherapy and sites of lesions (face vs trunk vs limbs) as we believe that the deckling incision will offer the most benefit in those with previous radiotherapy and in lesions on the face.

### Randomization Procedure

Following suitability for enrolment into the study and allocation of study code, the participants will be randomized to receive either the deckled incision or the standard straight line incision in a simple fashion using a random computer number generator. This will be done in the anesthetic bay after confirmation of patient suitability by checking the medical records. The surgeon will subsequently ring an external number and the study nurse will specify what treatment the individual lesions will receive.

### Adverse Events

An adverse event is any untoward medical occurrence that results in the following: death, is life-threatening, requires inpatient hospitalization or prolongation of existing hospitalization, persistent or significant disability and/or incapacity or congenital or birth defect, and any condition requiring medical or surgical intervention. An adverse event can therefore be any unfavorable or unintended sign, symptom, condition, and/or an observation that may or may not be related to the study treatment.

### Blinding

In order to achieve allocation concealment an external third party will be used to assign the treatment group. The surgeon will document in the patient notes which incision was carried out for a specific lesion. This will be entered into the patient database by an independent third party.

The patient will be blinded to the type of incision that is done for any given lesion. However, it is impractical to blind the individual surgeon. In addition, the outcome adjudicators will also be blinded to the incision used. The data collectors and analysis will be centralized and remain independent of the outcome adjudicators at all times. Medical record numbers will also be recorded to enable tracking of progress over time.

### Statistical Consideration

Sample size was calculated on the basis of a type I error of 5% (ie, *P* <.05) and a type II error of 10% (ie, power of 90%) with the aid of a statistician. Using formal sample size calculations we estimated a sample size of 250 patients for each treatment group. We anticipate an attrition rate of 10%, and subsequently, have increased our sample size by that amount for each treatment group.

Statistical analysis will be carried out by an external statistician. We intend to use independent two-sample *t* tests on primary endpoints and both chi-squared and independent two-sample *t* tests on secondary endpoints.

### Confidentiality, Storage and Archiving

The patients will be allocated a study code and their data will be de-identified. Following completion of the study the data will be kept on a secure server for a minimum of 15 years in the Department of Plastics and Reconstructive Surgery.

## Results

The study is expected to begin enrolment in August 2016. We anticipate that deckling will have superior scar outcomes when compared to the straight line incisions. From clinical experience this is especially true for lesions involving the face and in those areas of the skin that have undergone radiation therapy.

The study will be funded by the Plastics and Reconstructive Surgery Department at St Vincent’s Hospital, Sydney, Australia. Ethics approval has been obtained for the study.

## Discussion

The deckling study is a novel method for improving post-operative scar outcomes. Although this study mainly evaluates scar outcomes following excision of smaller lesions in plastic surgery procedures, its results can be applicable to a broad range of surgical procedures involving the skin. The learning curve associated with a new surgical procedure or technique can limit its uptake by surgeons. The deckling incision is simple to master with a minimal learning curve and has a small impact on the overall operating time.

### Conclusion

The deckling incision is a unique, simple and cost effective technique for improving post-operative scar outcomes.

## Abbreviations

POSAS: Patient and Observer Scar Assessment Scale
RCT: randomized controlled trial

## Appendix

Multimedia Appendix 1 Patient and Observer Scar scale - Observer.

Multimedia Appendix 2 Patient and Observer Scar Scale - Patient.
