# Supplementary material for: The Deckled Incision: Study Protocol for a Randomized Controlled Trial
Source: JMIR Res Protoc. 2016 Jul 12;5(3):e97. doi: 10.2196/resprot.5491 (PMC4961880; doi:10.2196/resprot.5491)
Supplement: Multimedia Appendix 1 [file resprot_v5i3e97_app1.pdf]

POSAS Observer scale

The Patient and Observer Scar Assessment Scale v2.0 / EN

Date of examination:

Observer:

Location:

Research / study:

Name of patient:

Date of birth:

Identification number:

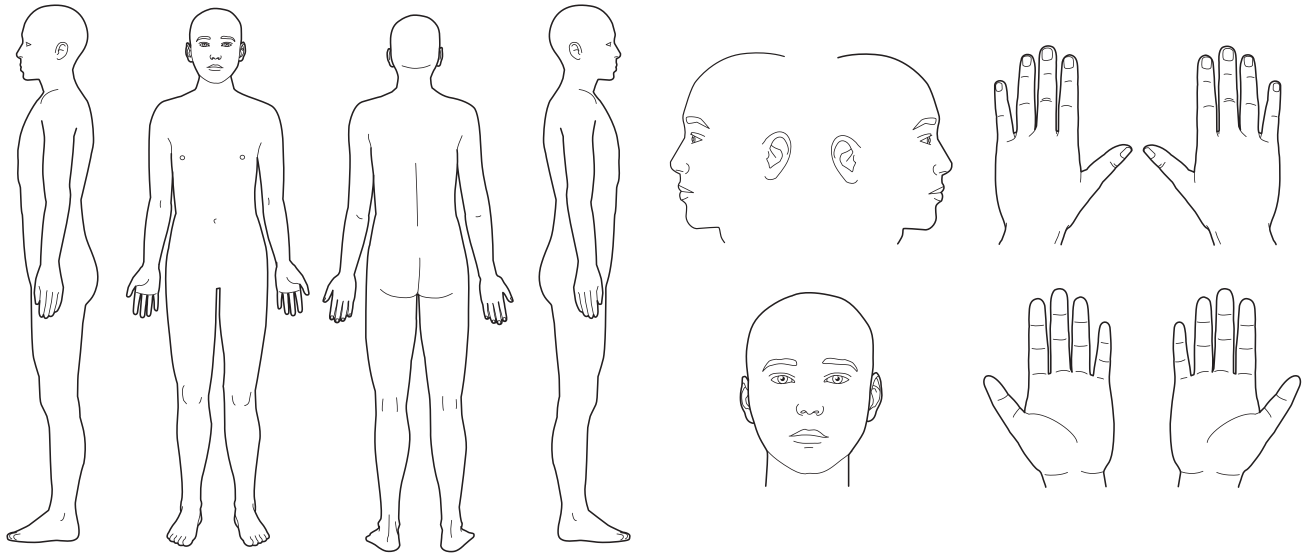

1 = normal skin | worst scar imaginable = 10

PARAMETER	1	2	3	4	5	6	7	8	9	10	CATEGORY
VASCULARITY											PALE / PINK / RED / PURPLE / MIX
PIGMENTATION											HYPO / HYPER / MIX
THICKNESS											THICKER / THINNER
RELIEF											MORE / LESS / MIX
PLIABILITY											SUPPLE / STIFF / MIX
SURFACE AREA											EXPANSION / CONTRACTION / MIX
OVERALL OPINION											

Explanation

The observer scale of the POSAS consists of six items (vascularity, pigmentation, thickness, relief, pliability and surface area).
All items are scored on a scale ranging from 1 ('like normal skin') to 10 ('worst scar imaginable').
The sum of the six items results in a total score of the POSAS observer scale. Categories boxes are added for each item. Furthermore, an overall opinion is scored on a scale ranging from 1 to 10.
All parameters should preferably be compared to normal skin on a comparable anatomic location.

Explanatory notes on the items:

- **VASCULARITY** Presence of vessels in scar tissue assessed by the amount of redness, tested by the amount of blood return after blanching with a piece of Plexiglas
- **PIGMENTATION** Brownish coloration of the scar by pigment (melanin); apply Plexiglas to the skin with moderate pressure to eliminate the effect of vascularity
- **THICKNESS** Average distance between the subcutical-dermal border and the epidermal surface of the scar
- **RELIEF** The extent to which surface irregularities are present (preferably compared with adjacent normal skin)
- **PLIABILITY** Suppleness of the scar tested by wrinkling the scar between the thumb and index finger
- **SURFACE AREA** Surface area of the scar in relation to the original wound area

COPYRIGHT © P.P.M. VAN ZUIJLEN, BEVERWIJK-NL
