# Supplementary material for: The Deckled Incision: Study Protocol for a Randomized Controlled Trial
Source: JMIR Res Protoc. 2016 Jul 12;5(3):e97. doi: 10.2196/resprot.5491 (PMC4961880; doi:10.2196/resprot.5491)
Supplement: Multimedia Appendix 2 [file resprot_v5i3e97_app2.pdf]

POSAS Patient scale

The Patient and Observer Scar Assessment Scale v2.0 / EN

Date of examination:

Observer:

Location:

Research / study:

Name of patient:

Date of birth:

Identification number:

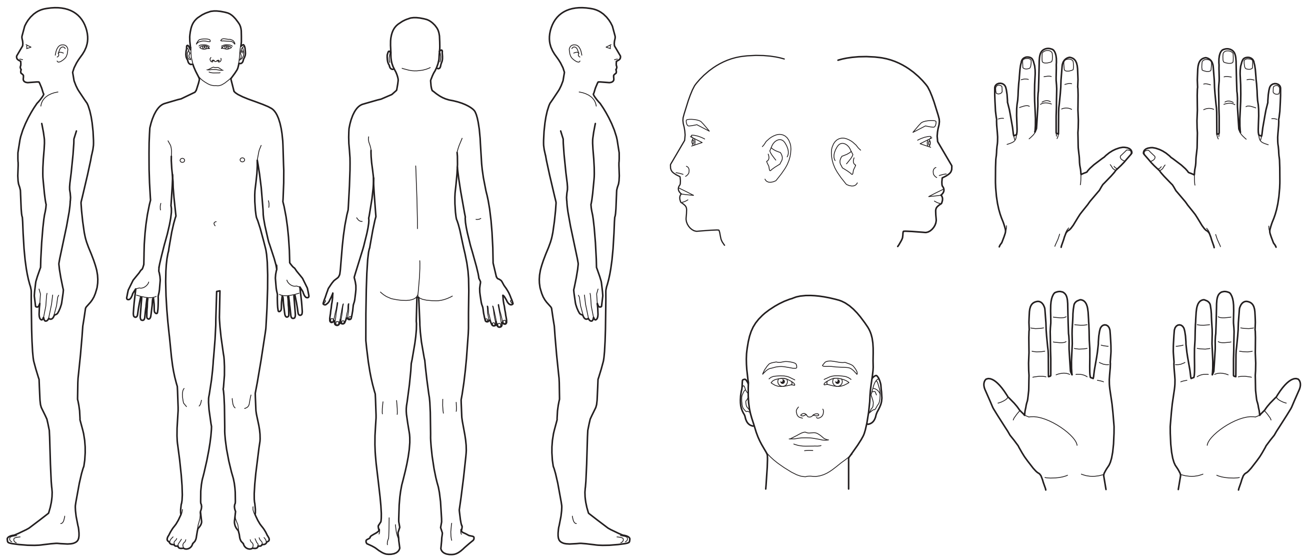

1 = no, not at all / yes, very much = 10	1	2	3	4	5	6	7	8	9	10
HAS THE SCAR BEEN PAINFUL THE PAST FEW WEEKS?										
HAS THE SCAR BEEN ITCHING THE PAST FEW WEEKS?										

1 = no, as normal skin / yes, very different = 10	1	2	3	4	5	6	7	8	9	10
IS THE SCAR COLOR DIFFERENT FROM THE COLOR OF YOUR NORMAL SKIN AT PRESENT?										
IS THE STIFFNESS OF THE SCAR DIFFERENT FROM YOUR NORMAL SKIN AT PRESENT?										
IS THE THICKNESS OF THE SCAR DIFFERENT FROM YOUR NORMAL SKIN AT PRESENT?										
IS THE SCAR MORE IRREGULAR THAN YOUR NORMAL SKIN AT PRESENT?										

1 = as normal skin / very different = 10	1	2	3	4	5	6	7	8	9	10
WHAT IS YOUR OVERALL OPINION OF THE SCAR COMPARED TO NORMAL SKIN?										

COPYRIGHT © P.P.M. VAN ZUIJLEN, BEVERWIJK-NL
